# Depression, Anxiety, Stress and Anger of the Cabin Crew during the COVID-19 Pandemic in South Korea

**DOI:** 10.3390/healthcare10101952

**Published:** 2022-10-06

**Authors:** Yeo-Won Jeong, Jung-Ha Kim

**Affiliations:** 1 Department of Nursing, College of Nursing, Dongguk University, Gyeongju-si 38066, Korea; 2 Aeromedical Center of Korean Air, Seoul 07505, Korea

**Keywords:** depression, anger, anxiety, flight attendant, psychological

## Abstract

This study examines the levels of depression, anxiety, stress, and state and trait anger of cabin crews, as well as the differences in the main variables and general characteristics between the depression and anxiety groups, during the COVID-19 pandemic. This descriptive cross-sectional study used data from 161 Korean cabin crew members. Data were analyzed using descriptive analysis, independent t-tests, and chi-square tests. Of the participants, 62.7% were women, and 52.2% and 46.6% were classified into the depression and anxiety groups, respectively. No differences in the general characteristics between the non-depression and depression groups were found. However, in the anxiety group, there were significant differences in age, marital status, position, and work type during the COVID-19 pandemic. Additionally, the anxiety group showed a higher anger state (approximately 6.76 times higher than the normal group) than the depression group (approximately 4.90 times higher than the normal group). In a pandemic, airlines should screen cabin crews for depression and anxiety. The development and applications of mental health education for high-risk groups should include anger intervention.

## 1. Introduction

From 2020 to the present, coronavirus disease 2019 (COVID-19) has caused a worldwide pandemic. In South Korea, a total of 620,938 cases were reported by December 2021 since the first case was reported in January 2020; among them, the proportion of imported cases from foreign countries was approximately 3% [1]. Because of the COVID-19 pandemic, worldwide travel, tourism, and leisure has been suspended. Various health communication strategies in each country, such as social distancing, the prohibition of travel and movement, and public facility closures, have been implemented [2]. In particular, as international flights and the number of passengers are considered important risk factors for the spread of COVID-19 [3], airlines decreased the number of flight operations and flight working days of the cabin crew, which affects the mental health of the cabin crew [4].

Cabin crew members who work abroad are at a higher risk of infection than other occupational groups. Moreover, there have been reports of cases where the cabin crew contracted COVID-19 while on flight duty [5]. To prevent the infection of the cabin crew, most airlines require them to wear face shields, N95 masks, gloves, or gowns while in-flight and are making efforts to minimize the risk of exposure to infection by simplifying in-flight services [6].

However, cabin crews are also responsible for passenger safety, such as implementing first aid on the aircraft; therefore, if a passenger is with suspected to have COVID-19 on flight, first aid should be provided by the cabin crew [7]. Although cabin crew members have a high awareness of and performance in infection prevention activities, they may be insufficient because they are non-medical personnel [8]. It has been reported that the cabin crew on flights experience a significant increase in levels of depression, stress, and anxiety that they may become infected [4]. In addition, during the COVID-19 period, levels of anxiety and depression among infected personnel, health-care workers, and the general public increased [9], as well as anger [10]. However, during the COVID-19 pandemic, studies on the anger of cabin crews who were exposed to the risk of infection are insufficient.

Furthermore, as many countries closed their borders because of COVID-19, most cabin crew members are on long-term leave, causing employment instability. A study has been conducted on the levels of depression, stress, and anxiety of the cabin crew when they had reduced work during the COVID-19 pandemic [4]; however, to the best of our knowledge, no study has been conducted on the cabin crew in South Korea. The cabin crew experience mental health problems, such as anxiety, depression, and anger regarding COVID-19 because of overseas work and stress owing to employment instability; hence, mental health management is urgent.

Therefore, this study aims to investigate the levels of depression, anxiety, stress, and anger of the cabin crew and the differences in the main variables between the depression and anxiety groups of the cabin crew during the COVID-19 pandemic to provide basic data for the screening of high-risk groups for depression and anxiety according to the general characteristics of the cabin crew and the foundation for the development of customized interventions.

## 2. Materials and Methods

### 2.1. Study Design and Participants

This study used a cross-sectional design to evaluate the levels of depression, stress, and anxiety of the cabin crew and the differences in the main variables and general characteristics between the depression and anxiety groups of the cabin crew during the COVID-19 pandemic. The inclusion criteria for the participants were as follows: (a) cabin crew working for airlines in South Korea; and (b) those who had more than one month of experience of flight duty. A total of 161 cabin attendants were included in the analysis.

### 2.2. Methods

#### 2.2.1. Depression and Anxiety

The short version of the Depression, Anxiety, and Stress Scale 21 (DASS-21) [11] was used. This scale includes 21 items with three subcategories (seven items each for depression, anxiety, and stress). Each item is rated on a four-point Likert scale, ranging from 0 (“Did not apply to me at all”) to 3 (“Applied to me very much or most of the time”). Higher scores indicated higher levels of depression or anxiety. In this study, the Korean version of the DASS-21 was used [12]. In the Korean version of the DASS-21, Cronbach’s alpha of depression and anxiety were 0.81 and 0.83, respectively [12]. In this study, Cronbach’s alpha of depression and anxiety were 0.918 and 0.921, respectively.

#### 2.2.2. Stress

Stress was measured using the Global Assessment of Recent Stress (GARS), developed by Linn (1986) and modified and adapted to Korean by Koh and Park (2000). The scale comprises eight items regarding stress levels in the previous week, and each item is rated on a 10-point Likert scale, ranging from 0 (none at all) to 10 (extreme). The total score ranges from 0 to 72, with higher scores indicating higher levels of stress. Cronbach’s alpha of the original scale was 0.69–0.92 [13], 0.86 of the Korean version [14], and 0.923 in the present study.

#### 2.2.3. Anger

We used the Korean version of the State–Trait Anger Expression Inventory (STAXI), developed by Spielberger (1988) and translated and validated by Chon, Hahn, and Lee (1998). The scale comprises 44 items, with 10 items on state anger (how people feel currently), 10 on trait anger (how people feel normally), and 24 on anger expression. State and trait anger were used in this study. State anger refers to the intensity of anger at a specific time, and temperament anger refers to the anger tendency of how often an individual feels anger [15]. Each item was rated on a four-point Likert scale, ranging from 0 (not at all) to 3 (very much so), and the total score ranged from 0 to 30, with higher scores indicating higher anxiety. Cronbach’s alpha for the state and trait anger were 0.93 and 0.86, respectively, in Spielberger’s (1988) study and 0.89 and 0.82, respectively, in the Korean version [16]. Cronbach’s alpha for the state and trait anger in this study were 0.948 and 0.949, respectively.

#### 2.2.4. General Characteristics

Sociodemographic variables included age, sex, marital status, resident status, education, employment type, work experience, position, work type at the time of the COVID-19 pandemic, work on an international flight during the COVID-19 pandemic, and protective equipment to be worn while in-flight. Marital status was classified as married or never married, and resident status was divided into living with others (family, spouse, or friends) or alone. Education was classified as university, college, or higher. Employment types were divided into regular, non-regular workers, and others. Work types during the COVID-19 pandemic were classified as continuous working, intermittent working, unpaid leave, and paid leave. Protective equipment to be worn while in-flight included KF94 or medical masks, gloves, goggles, full body protective clothing, and protective overshoes, and this was divided into one or more.

#### 2.2.5. Data Collection and Ethical Consideration

This study was conducted after obtaining approval from the institutional review board of the authors’ institution (DGU IRB 20210003). The study was conducted using a self-report online survey of participants who voluntarily agreed to participate in the survey, targeting cabin attendants working at three airlines in Korea between 21 August and 14 September 2021. The first online survey screen included the study background, purpose, and procedure. Furthermore, information for voluntary research, withdrawal from the study, and confidentiality was also included. The online survey was designed to show the click button for informed consent after reading the information of the study before displaying the questionnaire. The survey took approximately 15 min to complete, and when the participants’ contact information was entered at the end of the study, a mobile coupon was provided as a gift.

#### 2.2.6. Data Analysis

Data were analyzed using SPSS 25.0 (IBM Corp., Armonk, NY, USA). Participants’ characteristics and main variables were analyzed using descriptive statistics. In addition, t-test and chi-square tests were used to analyze the differences in the general characteristics and main variables according to the depression and anxiety groups. The depression (more than 10 total depression subscale scores on the DASS 21) and anxiety groups (more than 8 total anxiety subscale scores on the DASS 21) followed the standard cut-off scores of the DASS 21, as suggested by Lovibond and Lovibond (1995). Correlations among the main variables were analyzed using Pearson’s correlation coefficients. Statistical significance was set at *p* < 0.05.

## 3. Results

### 3.1. General Characteristics

Of the 161 participants, 101 (62.7%) were women and 114 (70.8%) were married (Table 1). The number of years of work experience was 12.98 ± 6.97, and 75.2% of the participants were pursers and team leaders. During the COVID-19 pandemic, 114 (70.8%) of the participants were working, and 36 (22.4%) wore less than three pieces of protective equipment (KF94 masks, gloves, or goggles) while on duty. On the other hand, 125 (77.6%) of the participants wore more than three pieces of protective equipment (KF94s, gloves, goggles, protective clothing, or protective shoes).

### 3.2. Level and Correlation among the Main Variables

The mean scores for depression, anxiety, and stress were 6.07 ± 5.25, 4.63 ± 4.94, and 33.05 ± 16.65, respectively (Table 2). The mean scores for state and trait anger were 3.82 ± 5.81 and 9.90 ± 8.08, respectively. Depression was positively correlated with anxiety (*r* = 0.823, *p* <.000), stress (*r* = 0.666, *p* <.000), state anger (*r* = 0.562, *p* <.000), and trait anger (*r* = 0.461, *p* <.000).

### 3.3. Differences in the General Characteristics and Main Variables in the Depression and Anxiety Groups

Of the participants, 52.2% (84) were assigned to the depression group and 46.6% (75) were assigned to the anxiety group. No difference was found in the general characteristics between non-depression and depression groups. In the anxiety group, there were significant differences in age (*t* = −2.293, *p* = 0.023), marital status (χ^2^ = 4.196, *p* = 0.041), position (χ^2^ = 5.883, *p* = 0.015), and work type (χ^2^ = 7.934, *p* = 0.005) during the COVID-19 outbreak. As shown in Table 3, the depression group had higher anxiety and stress levels than the non-depression group (*t* = −10.64, *p* < 0.001 and *t* = −7.95, *p* < 0.001, respectively). In addition, the depression group had higher state and trait anger levels than the non-depression group (*t* = −5.90, *p* < 0.001 and *t* = −6.24, *p* < 0.001, respectively). The anxiety group had higher levels of depression, stress, state anger, and trait anger than the non-anxiety group (*t* = −12.26, *p* < 0.001; *t* = −8.20, *p* < 0.001; *t* = −7.56, *p* < 0.001; and *t* = −7.56, *p* < 0.001, respectively).

## 4. Discussion

We assessed the levels of depression, anxiety, stress, and anger in cabin crews during the COVID-19 pandemic. This study is significant as it is the first to identify psychological problems in South Korean cabin crew members during the COVID-19 pandemic.

The anxiety level was 4.6, which was higher than that of a previous study [4]. This may have been due to the timing of data collection. While our study was conducted in 2021, which was approximately two years after the COVID-19 pandemic in Korea, a previous study was conducted in 2020. Cabin crew members experienced more salary reductions during these two years. This may have caused anxiety [17]. Another reason for this is that there were more cabin crew members who were still on flight duty than in the previous study. In this study, approximately 71% of the cabin crew members were still on flights; however, only 13% of the cabin crew members were still on flights in a previous study. This may be related to concerns that they may have been infected with COVID-19 during their flight duty. However, most Korean airlines attempt to allocate flight duty work and leave periods equally for all their cabin crew. Therefore, even if they were on flight duty during the data collection period, they may also be on leave. This might increase anxiety levels because of the changed flight work pattern, rather than the flight-duty work [18].

We found that the participants’ age in the anxiety group was higher; however, there was no difference in the participants’ age in the depressed group. This result differed from that of a previous study, in which younger respondents had higher levels of depression and anxiety [4]. **This** may be related to a higher age of the respondents in the previous study. The average age of participants in the previous study was 31 years, while the average years of participants in this study is 38 years. In the case of older respondents, the level of anxiety in married respondents may be higher than that of younger respondents; therefore, married respondents may have been more anxious that the virus could spread to their families. Moreover, we found that approximately 52% of married respondents belonged to the anxiety group. Another reason for this is that the respondents were aware that older people had a higher mortality rate after COVID-19 infection [19]. In South Korea, 94% of those infected with COVID-19 are over the age of 60 years [1].

The anxiety level of team leaders was higher than that of the cabin crew staff. There were several reasons for this. First, the higher the position, the higher the anxiety level [20]. Most Korean airlines need to decrease the flight-duty period of their cabin crew because of travel bans and border closures in other countries. Therefore, cabin crews might be concerned about the possibility of returning from flight duty and being dismissed. In the case of team leaders with high salaries, airlines may consider them to be a priority for dismissals [20]. Furthermore, the burden of checking the immigration regulations of various countries, which change frequently, may have contributed to anxiety. Because cabin crews work as a team during flights, team leaders should be more familiar with the regulations than other cabin crew members. Finally, this may be due to the in-flight working process. When patients have a suspected infectious disease, the team leader handles them directly or instructs other cabin crew members according to the airline policy [8].

In this study, the levels of depression, anxiety, stress, and state and trait anger in the depression or anxiety groups were higher than those in the non-depression or anxiety groups during the COVID-19 pandemic. In particular, comparing the difference within the groups using the main variable, the state anger of the depression group was approximately 4.9 times higher than that of the non-depression group, and the state anger of the anxiety group was approximately 6.76 times higher than that of the non-anxiety group. In other words, the anxiety group had a higher anger level than the depression group, which is similar to previous studies [21,22].

Studies have reported that generalized anxiety disorder includes anger symptoms as a diagnostic criterion, and anger is elevated in groups with anxiety-related disorders [23,24]. In addition, several aspects can explain the high level of anger in the cabin crew of the anxious group during the COVID-19 period. First, the high anger level of the anxiety group among cabin crews is due to various restrictions on social activities, such as social distancing and isolation [21]. Furthermore, the Incheon quarantine station isolated cabin crews in charge of a patient with suspected or confirmed COVID-19 during a flight. The flight-duty schedule was decided on a monthly basis owing to the closure of the airline, but approximately 50% of flight duties were carried out during a quarantine period, which may have caused anger and anxiety. A previous study reported that approximately 11% of Korean cabin crews experienced quarantine because of contact with suspected and confirmed cases of COVID-19 [8]. In addition, when a cabin crew stays abroad for flight duty, many countries and airlines require them to not leave the hotel and eat alone in their hotel rooms [6].

The results suggest that airlines should measure the levels of depression and anxiety of cabin crews during the COVID-19 pandemic, and that it is necessary to develop and apply a mental health program that includes content on how to recognize anger and effectively express and deal with anger for high-risk groups. Furthermore, airlines should develop strategies to decrease anxiety, such as psychological counselling for team leaders and married cabin crew members.

This study has several limitations and recommendations for future studies. First, as this is not a longitudinal study, it is difficult to confirm whether the psychological problems of the cabin crew changed after the COVID-19 pandemic. A longitudinal study should be proposed in the future. Second, the study should be carefully interpreted based on the study results of Korean airlines. Finally, we suggest a study on coping strategies for the mental health management of cabin crews during an infectious disease pandemic.

## 5. Conclusions

In this study, the cabin crews demonstrated high anxiety levels during the COVID-19 pandemic. Additionally, the study found that age and position were related to anxiety levels. In an infectious disease pandemic, airlines should screen cabin crews for depression and anxiety, and when developing and applying mental health education for high-risk groups, it is necessary to include anger intervention.

## Figures and Tables

**Table 1:** General characteristics.

Variables	Total n (%) or M ± SD	Range
Age (year)	37.92 ± 6.32	25.0–60.0
Sex		
Male	60 (37.3)	
Female	101 (62.7)	
Marital status		
Married	114 (70.8)	
Never married	47 (29.2)	
Resident status		
Living with others	135 (83.9)	
Living alone	26 (16.1)	
Education		
University or college	148 (91.9)	
Higher	13 (8.1)	
Employment type		
Regular worker	157 (97.5)	
Non-regular worker and others	4 (2.5)	
Work experience (years)	12.98 ± 6.97	2–34.8
Position		
Purser	121 (75.2)	
Cabin attendant	40 (24.8)	
Work type during the COVID-19 pandemic		
Working	114 (70.8)	
Leave (paid or unpaid)	47 (29.2)	
Work on an international flight during the COVID-19 pandemic		
Yes	133 (82.6)	
No	28 (17.4)	
Number of protective equipment worn during flight		
Less than three	36 (22.4)	
More than three	125 (77.6)	

**Table 2:** Level and correlation among the main variables.

Variables	M ± SD	Range	1	2	3	4	5
1. Depression	6.07 ± 5.25	0–20	1				
2. Anxiety	4.63 ± 4.94	0–21	0.823 **	1			
3. Stress	33.05 ± 16.65	0–72	0.666 **	0.620 **	1		
4. State anger	3.82 ± 5.81	0–24	0.562 **	0.624 **	0.463 **	1	
5. Trait anger	9.90 ± 8.08	0–30	0.461 **	0.524 **	0.531 **	0.652 **	1

** *p* < 0.001.

**Table 3:** Differences in the general characteristics and main variables in the depression and anxiety groups.

Variables	Depression			Anxiety		
	Non-Depression Group (n = 77)	Depression Group (n = 84)		Non-Anxiety Group (n = 86)	Anxiety Group (n = 75)	
	Total n (Column %) or M ± SD	Total n (Column %) or M ± SD	t or χ^2^ (*p*)	Total n (Column %) or M ± SD	Total n (Column %) or M ± SD	t or χ^2^ (*p*)
**General characteristics**						
Age (year)	370.38 ± 70.13	380.43 ± 50.48	−10.04 (0.299)	360.82 ± 60.78	390.13 ± 50.56	−20.293 * (0.023)
Sex						
Male	30 (500.5)	30 (500.0)	0.181 (0.670)	31 (510.7)	29 (480.3)	00.118 (0.732)
Female	47 (390.0)	54 (530.5)		55 (540.5)	46 (450.5)	
Marital status						
Married	49 (430.0)	65 (570.0)	30.672 (0.055)	55 (480.2)	59 (510.8)	40.196* (0.041)
Never married	28 (590.6)	19 (400.4)		31 (660.0)	16 (340.0)	
Resident status						
Living with others	63 (460.7)	72 (530.3)	00.450 (0.502)	69 (510.1)	66 (480.9)	10.785 (0.182)
Living alone	14 (530.8)	12 (460.2)		17 (650.4)	9 (340.6)	
Education						
University or college	70 (470.3)	78 (520.7)	00.205 (0.650)	79 (530.4)	69 (460.6)	00.001 (0.974)
Higher	7 (530.8)	6 (460.2)		7 (530.8)	6 (460.2)	
Employment type						
Regular worker	76 (480.4)	81 (510.6)	00.857 (0.622)	85 (540.1)	72 (450.9)	10.331 (0.260)
Non-regular worker and others	1 (250.0)	3 (750.0)		1 (250.0)	3 (750.0)	
Work experience (years)	120.55 ± 70.67	130.39 ± 60.30	−00.769 (0.443)	120.16 ± 70.21	130.94 ± 60.62	−10.631 (0.105)
Position						
Purser	53 (430.8)	68 (560.2)	30.161 (0.075)	58 (470.9)	63 (520.1)	50.883 * (0.015)
Cabin attendant	24 (600.0)	16 (400.0)		28 (700.0)	12 (300.0)	
Work type during the COVID−19 pandemic						
Working	59 (510.8)	55 (480.2)	20.415 (0.120)	69 (600.5)	45 (390.5)	70.934 * (0.005)
Leave (paid or unpaid)	18 (380.3)	29 (610.7)		17 (360.2)	30 (630.8)	
Work on an international flight during the COVID−19 pandemic						
Yes	63 (470.4)	70 (520.6)	00.064 (0.800)	68 (510.1)	65 (480.9)	10.609 (0.205)
No	14 (500.5)	14 (500.5)		18 (640.3)	10 (460.6)	
Number of protective equipment to be worn during flight						
Less than three	18 (500.5)	18 (500.5)	00.088 (0.767)	21 (580.3)	15 (410.7)	00.451 (0.502)
More than three	59 (470.2)	66 (520.8)		65 (520.0)	60 (480.0)	
**Main variables**						
Depression	10.51 ± 10.37	100.26 ± 30.80	−190.09 **	20.66 ± 20.95	90.99 ± 40.55	−120.26 **
Anxiety	10.31 ± 10.69	70.68 ± 40.99	−100.64 **	00.93 ± 10.05	80.89 ± 40.18	−170.06 **
Stress	230.81 ± 140.30	410.54 ± 130.98	−70.94 **	240.61 ± 140.49	420.75 ± 130.44	−80.24 **
State anger	10.26 ± 20.93	60.18 ± 60.76	−50.90 **	10.04 ± 20.42	70.03 ± 60.86	−70.56 **
Trait anger	60.17 ± 50.76	130.33 ± 80.42	−60.24 **	60.04 ± 50.77	140.35 ± 80.12	−70.56 **

* *p* < 0.05, ** *p* < 0.001.

## Data Availability

Not applicable.
